# Head-on collision and overtaking collision between an envelope solitary wave and a KdV solitary wave in a dusty plasma

**DOI:** 10.1038/srep21214

**Published:** 2016-02-12

**Authors:** Heng Zhang, Wen-Shan Duan, Xin Qi, Lei Yang

**Affiliations:** 1 College of Physics and Electronic Engineering and Joint Laboratory of Atomic and Molecular Physics of Northwest Normal University, Lanzhou 730070, China; 2 Institute of Modern Physics, Chinese Academy of Sciences, Lanzhou 730000, China; 3 Department of Physics, Lanzhou University, Lanzhou 730000, China

## Abstract

Head-on collision and overtaking collision between a KdV solitary wave and an envelope solitary wave are first studied in present paper by using Particle-in-cell (PIC) method in a dusty plasma. There are phase shifts of the KdV solitary wave in both head-on collision and the overtaking collision, while no phase shift is found for the envelop solitary wave in any cases. The remarkable difference between head-on collision and the overtaking collision is that the phase shift of KdV solitary wave increases as amplitude of KdV solitary wave increases in head-on collision, while it decreases as amplitude of the KdV solitary wave increases in the overtaking collision. It is found that the maximum amplitude during the collision process is less than sum of two amplitudes of both solitary waves, but is larger than either of the amplitude.

The field of plasma physics is rapidly growing due to its extensive application in space plasmas^1^,^2^,^3^,^4^, microwave transmision^5^,^6^, fusion plasmas^7^,^8^,^9^,^10^ and so on. Plasmas containing dust particles are called the dusty plasmas which are also extensively studied because of their relevance in many spaces and technological applications^11^,^12^,^13^. Meanwhile dusty plasma supports a variety of collective modes and nonlinear coherent structures. The waves in a dusty plasma have been a interesting topic for many years. The existence of dust acoustic wave (DAW) was first theoretically predicted by Rao *et al.*^14^ in an unmagnetized plasma consisting of the inertial dust fluid, Boltzmann distributed electrons and ions. Since then, the DAW has been extensively studied analytically^15^,^16^,^17^,^18^ and verified by the experiments^19^,^20^,^21^. As well known, this kind of nonlinear wave can be described by either a Korteweg-de-Vries (KdV) equation or a nonlinear Schrödinger equation (NLSE). The KdV equation describes evolution of a unmodulated wave, called KdV soliton. Whereas, the NLSE usually describes the modulated waves. One of the solutions of the NLSE is called envelope soliton. Admin and Shukla *et al.*^22^ studied the modulational instability of the dust acoustic waves and the dust-ion-waves. Ghosh *et al.*^23^ studied the effects of the dust charge fluctuations of the low-frequency wave modulation.

The interaction between two KdV solitary waves is a subject of great interest^24^,^25^,^26^. In general, when two solitary waves propagate in one-dimensional plasmas, they can undergo two kinds of interaction^27^. One is the overtaking collision when both propagate in the same direction but with different propagation speed which can be investigated by the inverse scattering method^28^. The other is called the head-on collision when both propagate in opposite directions which is usually studied by the extended Poincare-Lighthill-Kuo (PLK) method^29^,^30^,^31^,^32^,^33^,^34^,^35^. The head-on collision between two KdV solitary waves have been verified from both the experiments^36^,^37^ and the numerical simulation by using the PIC simulation method^38^. Recently, the head-on collision between two envelope solitary waves are studied analytically^39^. The head-on collision between two envelope solitary waves has also been studied by using the PIC method^40^. Interesting results are found when head-on collision between two envelope solitary waves happen. There is a phase shift after a head-on collision between two KdV solitary waves^38^, while there is no phase shift between two envelope solitary waves^40^.

However, no report is given on collision between a KdV solitary wave and an envelope one. In the present paper we try to discuss this question by using the PIC simulation method. Both the head-on collision and overtaking collision between two kinds of waves will be studied in this paper.

It is found that there are phase shifts for a KdV solitary wave during the collision between KdV solitary wave and envelope solitary wave, which is similar to that between two KdV solitary waves in which there are phase shifts for both colliding solitary waves^38^. However, there is no phase shift for the envelope solitary wave after the collision.

## Results

### Analytical solutions of KdV and NLSE by using the perturbation method

The interaction between a KdV solitary wave and an envelope solitary wave in a dusty plasma will be studied in the present paper by using the one dimensional (1D) PIC method in an infinite background plasma. First we give the 1D dimensionless normalized equations of motion for system^38^,^40^


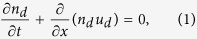



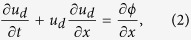



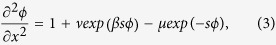


where *β* = *T*_*i*_/*T*_*e*_ is the ratio of the ion and electron temperatures, *s* = 1/(*μ* + *νβ*), *μ* and *ν* are the normalized ion and electron number densities, respectively, *n*_*d*_ and *u*_*d*_ refer to the density, the velocity of the dust grains respectively, *ϕ* is the electrostatic potential. In the simulation, we take *β* = 0.1, *μ* = 1.1 and *ν* = 0.1.

The spatial coordinate *x*, the time *t*, the velocity and the electrostatic potential *ϕ* are normalized by the Debye length *λ*_*D*_ = (*T*_*eff*_ /4*πZ*_*d*_*n*_*d*0_*e*^2^)^1/2^, the inverse of effective dust plasma frequency 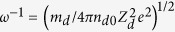, the dust acoustic speed *C*_*d*_ = (*Z*_*d*_*T*_*eff*_/*m*_*d*_)^1/2^ and *T*_*eff*_/*e*, respectively, where the effective temperature is defined as 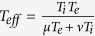.

By introducing the appropriate stretched coordinates *ξ* = *ε*(*x* − *ct*) and *τ* = *ε*^3^*t*, we can obtain a KdV equation as follows


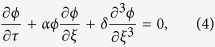


where 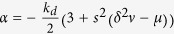, 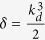. *k*_*d*_ and *c* are the wave number and velocity of the KdV solitary wave, respectively, where *k*_*d*_ = 1.0. One of the solution of Eq. (4) is 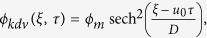 where 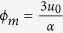, 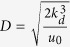, *u*_0_ = 1.0. We choose the same parameters as that in ref. 38.

NLSE can also be obtained as follows by using the different transformations^40^


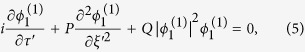


where *ξ*′ = *ε*(*x* − *u*_*s*_*t*), *τ*′ = *ε*^2^*t, u*_*s*_ is group velocity of envelope solitary wave. *P* and *Q* are functions of system parameters. In the simulation, we choose system parameters as follows: wave number of envelope wave *k*_*n*_ = 0.1, then we obtain: *P* = −0.146, *Q* = −3.65^40^. One of the envelope solitary wave solution of Eq. (5) is: 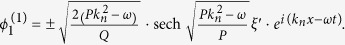 If the signs of both *ω* and *u*_*s*_ are positive, the envelope wave propagates in the positive *x* direction. Otherwise, the wave propagates in the negative *x* direction.

### 0.1 PIC simulation results of the head-on collision

In simulation, the initial conditions of the envelope solitary wave are given as follows: 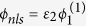, 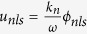 and 
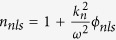
. *ϕ*_*nls*_, *u*_*nls*_ and *n*_*nls*_ are electrostatic potential, velocity and density of dust grains corresponding to envelope solitary wave, respectively. The initial conditions of the KdV solitary wave are also chosen from the analytical solution: 
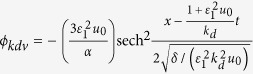
, *u*_*kdv*_ = *ϕ*_*kdv*_ and *n*_*kdv*_ = 1 + *ϕ*_*kdv*_. *ϕ*_*kdv*_, *u*_*kdv*_ and *n*_*kdv*_ are electrostatic potential, velocity and density of dust grains corresponding to KdV solitary wave, respectively.

The dust particles are represented as superparticles, while the ions and the electrons are modeled as Boltzmann distributed background. The area weighting technique is used to deposit the charge on the 2th nearest grid points in order to obtain the spatial distribution of the particles on the mesh. At the start of simulations, 100 superparticles with different weighting parameters are allocated symmetrically in each grid. Weighting parameters and initial velocities of superparticles are carefully chosen to make sure that the density and velocity distribution of dust particles are given as those obtained by the reductive perturbation method. The electric field is determined by the nonlinear Poisson-Boltzmann’s equation. The motion equations for superparticles are integrated by a standard leap-frog algorithm.

**Figure 1:**
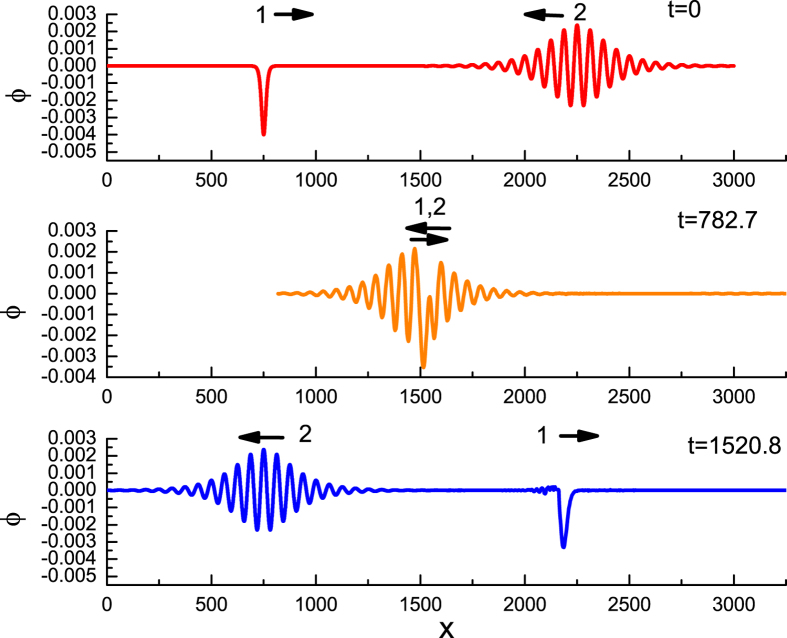
The evolution of head-on-collision of KdV solitary wave 1 and an envelope solitary wave 2 at the different times where *ε*_1_ = 0.002, *ε*_2_ = 0.01, *μ* = 1.1, *ν* = 0.1, *β* = 0.1.

**Figure 2:**
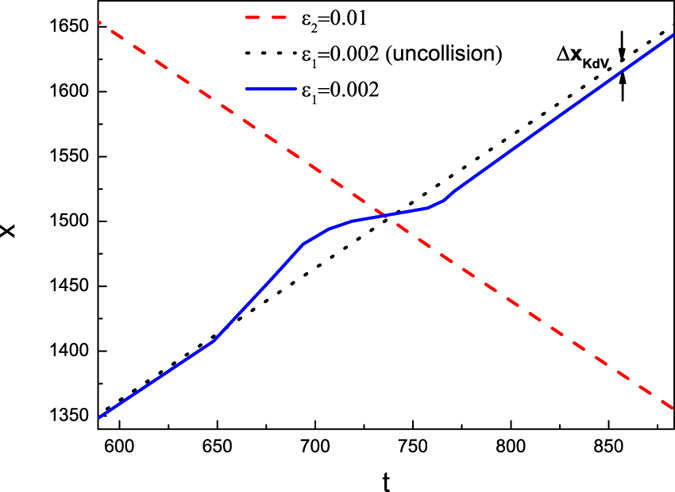
The trajectories of the head on collision between an envelope solitary wave 2 and a KdV solitary wave 1, where *ε*_1_ = 0.002, *ε*_2_ = 0.01, *μ* = 1.1, *ν* = 0.1, *β* = 0.1.

Initially the KdV solitary wave propagates in the positive *x* direction, while the envelope solitary wave propagates in the negative *x* direction, shown in Fig. 1. Both are far apart. After some time, they interact, collide, and then depart. It seems that the waveform and the propagation speeds of both solitary waves remain unchanged after the collision is over. The colliding process are clearly shown in both Figs 1 and 2.

**Figure 3:**
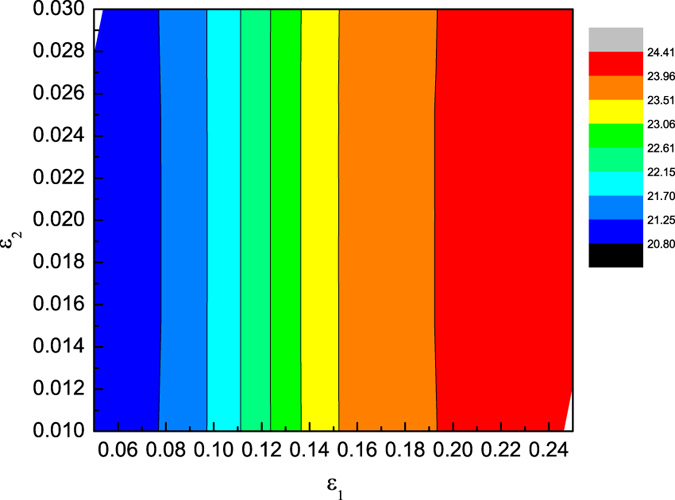
The phase delay of KdV solitary wave 1 after collision with envelop solitary wave 2 as a function of amplitudes of both *ε*_1_ and *ε*_2_.

In Fig. 2 the colliding trajectories are pictured. There are no phase shift of the envelope solitary wave, i.e., the propagation velocity of the envelope solitary wave is a constant. However, the propagation velocity of the KdV solitary wave is not a constant during the collision. During the collision, the propagation velocity of the KdV solitary wave first becomes larger, then smaller and finally become a constant as that of the previous one. Furthermore, a phase delay of KdV solitary wave is observed. The definition of the phase shift is shown in Fig. 2. In order to further understand the dependence of the phase shifts of both solitary waves on their amplitudes, the dependence of phase shifts of the KdV solitary waves on the parameters of both *ε*_1_ and *ε*_2_ are given in Fig. 3. It is noted that the phase shifts of KdV solitary waves only depend on its amplitudes, i.e., *ε*_1_, but not that of the envelope wave, i.e., *ε*_2_. Furthermore, It seems that there is no phase shift for the envelope solitary wave after the collision in any case.

**Figure 4:**
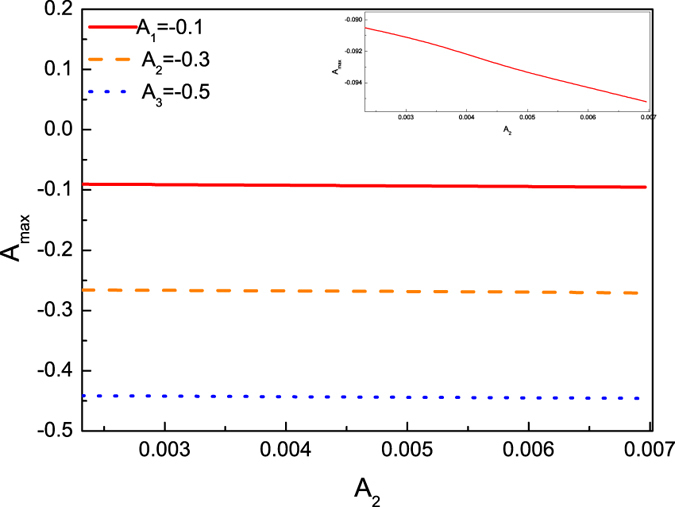
The dependence of the maximum amplitude *A*_*max*_ in colliding process on envelop solitary wave amplitudes *A*_2_ for different amplitudes of KdV solitary wave *A*_1_, *A*_2_ and *A*_3_.

It is observed that a maximum amplitude will be reached during the collision between two solitary waves. It indicates, however, that the maximum value is less than the sum of two amplitudes of both solitary waves, but it is larger than either of the amplitude. In order to understand this situation in detail, the dependence of the maximum amplitudes in the colliding process between two solitary waves on both of their initial amplitudes is shown in Fig. 4. It is noted that the maximum amplitude in colliding process increases as the amplitudes of both colliding solitary waves increase. It seems that the maximum amplitude is less than the sum of two amplitudes of both the KdV solitary wave and the envelop solitary wave.

### PIC simulation results of the overtaking collision

**Figure 5:**
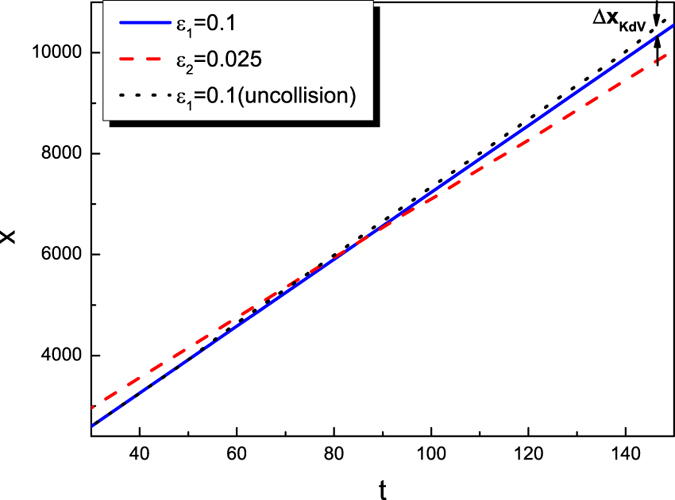
The trajectory of the overtaking collision between a KdV solitary wave 1 and an envelope solitary wave 2. The phase delay of the KdV solitary wave is observed, while no phase shift for an envelope solitary wave are found. The definition of the phase shift of the KdV solitary wave is shown in the figure.

**Figure 6:**
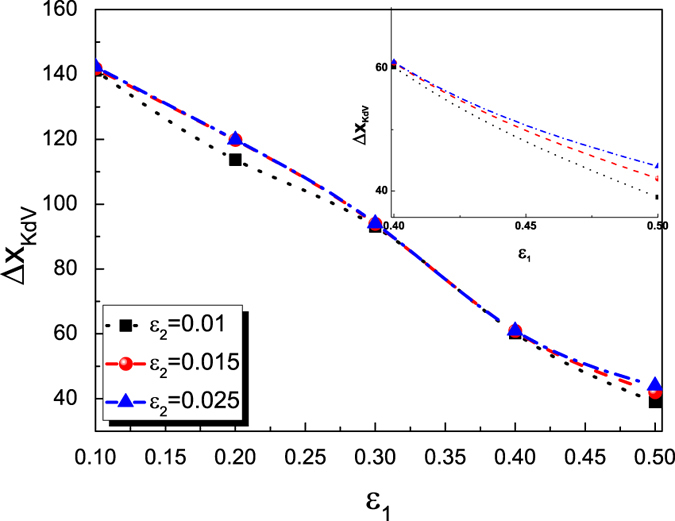
The dependence of the phase delay of the KdV solitary wave for the overtaking collision between an envelope solitary wave 2 and a the KdV solitary wave 1 on the amplitude of the KdV solitary wave.

**Figure 7:**
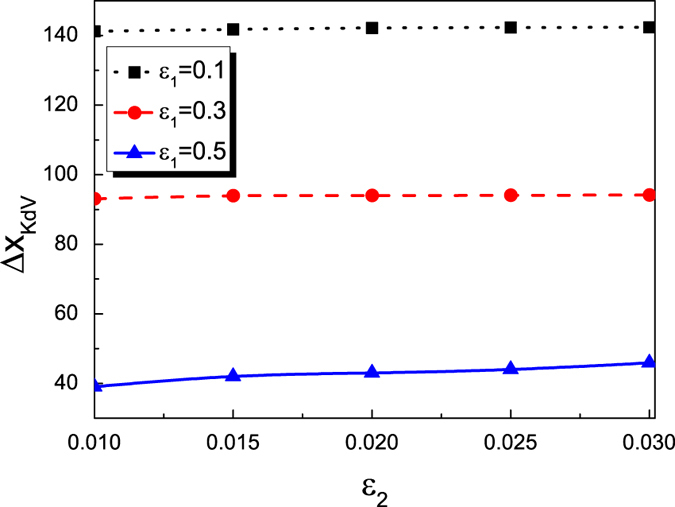
The dependence of the phase delay of the KdV solitary wave for the overtaking collision between an envelope solitary wave 2 and a the KdV solitary wave 1 on the amplitude of the envelope solitary wave.

When the overtaking collision between two solitary waves takes place, both solitary waves propagate in the same direction (positive *x* direction). In the simulations, the initial conditions of the envelope solitary wave is: 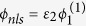, 
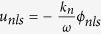
 and 
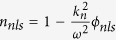
. The initial conditions of the KdV solitary wave is: 
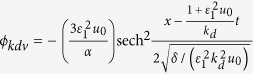
, *u*_*kdv*_ = *ϕ*_*kdv*_ and *n*_*kdv*_ = 1 + *ϕ*_*kdv*_. Because the propagation velocity of the envelope solitary wave is constant, while for the KdV solitary wave the propagation velocity depends on its amplitude. We choose the large amplitude KdV solitary wave in order that it can overtake the envelope solitary wave in a short period. The trajectories of the overtaking collision between two solitary waves are shown in Fig. 5. It is found that there is no phase shift for the envelope solitary wave after the overtaking collision is over, while there is phase shift for the KdV solitary wave. The dependence of the phase shifts of the KdV solitary waves on the parameter of *ε*_1_ characterizing the amplitude of the KdV solitary wave are given in Fig. 6. It indicates that the phase shift decreases as the amplitude of the colliding KdV solitary wave increases. Moreover, the dependence of the phase shift of the KdV solitary wave on the parameter of *ε*_2_ characterizing the amplitude of the envelope solitary wave is given in Fig. 7. It seems that the phase shift of the KdV solitary wave is almost independent of the amplitude of the envelope solitary wave. It is noted that the phase shift decreases as the amplitude of the KdV solitary wave increases in the overtaking collision which is different from that in head-on collision.

## Discussion

Both the head-on collision and overtaking collision between a KdV solitary wave and an envelope solitary wave are investigated by using PIC method in a dusty plasma. It is found that there are phase delay for KdV solitary wave for either the head on collision or the overtaking collision between a KdV solitary wave and an envelope solitary wave. It is observed that there are no phase delay for the envelop solitary wave in any cases. The remarkable difference between the head-on collision and the overtaking collision is that the phase shift of the KdV solitary wave increases as the amplitude of the KdV solitary wave increases in the head-on collision, while it decreases as the amplitude of the KdV solitary wave increases in the overtaking collision. It is found that the maximum amplitude during the collision process is less than the sum of two amplitudes of both solitary waves, but is larger than either of the amplitude. It is noted that the maximum amplitude in colliding process increases as the amplitudes of both colliding solitary waves increase. Meanwhile, PIC simulation provides a more realistic description of the dynamics of nonlinear dust acoustic waves that can be usefully applied to various low frequency phenomena observed in laboratory as well as space plasmas. The results has potential applications in the instability study of the space plasmas, and the fusion plasmas.

## Methods

**Figure 8:**
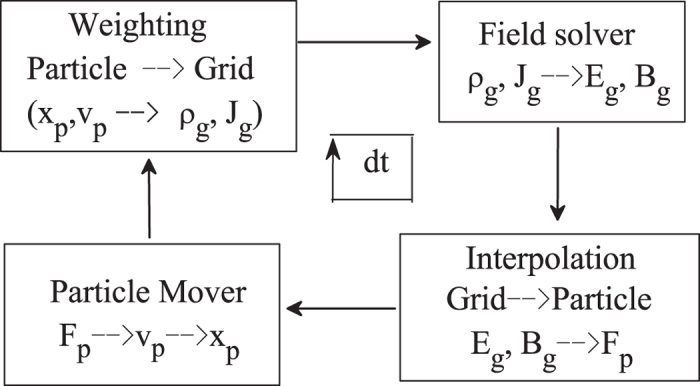
The summary of a computational cycle of the PIC method.

Numerical experiment is performed by using the one-dimensional PIC simulation method to study interaction between a KdV solitary wave and an envelope solitary wave in a dusty plasma in present work. During the simulation, The dust particles are represented as kinetic particles, while ions and electrons are modeled as Boltzmann distributed background. As well known, the real systems always contain very large amount of particles. In order to make simulations efficient or at least possible, so-called super-particles(SPs) are used. Each SP has a weight factor S specifying the number of real particles contained. Therefore, the equation of motion of the system is the Newton’s equation as follows


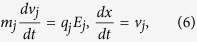


where *m*_*j*_, *q*_*j*_, *x*_*j*_ are the mass, charge and position of the *j*th SP, respectively. *E*_*j*_ is the electric field at the position of the *j*th SP. As the dust particles follow their trajectories, they continually exchange information with the background grid. Each dust particle contributes its charge to the corners of its instantaneous host cell. Therefore, the simulation region is divided to contain several grid cells during the PIC simulation. At each time step, the velocities, the positions of SPs are weighted to all the grids to calculate the charge density *ρ*_*g*_ (or electric current density *J*_*g*_). Once *ρ*_*g*_ is obtained, the Maxwell’s equations (electromagnetic model) or Poisson-Boltzmann equation (electrostatic model) will be solved numerically to derive the value of *E* at each grid. In electrostatic model, *B*_*g*_ = 0. Then the field imposed on each SP can be worked out and each SP will be driven by electric field according to Eq. (6), which will be solved numerically via the leap-frog algorithm. At last, the new positions and velocities are obtained, the procedure come to repeat until the simulation completed. The flowchart of PIC method is shown in Fig. 8.

In process of head-on collision between the KdV solitary wave and the envelope solitary waves, we assume that there are two opposite propagating solitary waves. the KdV solitary wave is propagating in the positive *x* direction and the envelop wave is in the negative *x* direction. The initial number density and the velocity of the dust particles are: 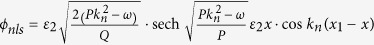, 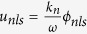 and 
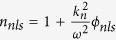
, where *x*_1_ is the initial phase of the envelope solitary wave. The initial condition of the KdV solitary wave is also chosen from the analytical solution, 
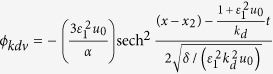
, *u*_*kdv*_ = *ϕ*_*kdv*_ and *n*_*kdv*_ = 1 + *ϕ*_*kdv*_, where *x*_2_ is the initial phase of the KdV solitary wave. In the simulation we choose *x*_1_ = 2250, *x*_2_ = 725 which ensure that two solitary waves are far enough. The parameters of two colliding waves are *ε*_1_ = 0.002, *ε*_2_ = 0.01, *k*_*d*_ = 1.0, *k*_*n*_ = 0.1, *μ* = 1.1, *ν* = 0.1, *β* = 0.1, *x*_1_ = *LX*/4, *x*_2_ = 3*LX*/4, Δ*x* = 0.1, Δ*t* = Δ*x*/100, *N*_*x*_ = 10000, while *ω* = 0.1, *u*_*s*_ = −0.985 and the number of super particles contained per cell is 100, where the negative sign stands for the envelop solitary wave propagating in the negative *x* direction.

In process of overtaking collision between KdV solitary wave and envelope solitary wave, both solitary waves propagate in same direction (positive *x* direction). The initial conditions are: 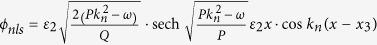, 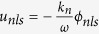 and 
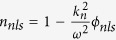
, where *x*_3_ is the initial phase of the envelope solitary wave. The initial condition of the KdV solitary wave is also chosen from the analytical solution, 
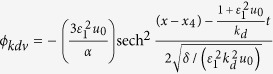
, *u*_*kdv*_ = *ϕ*_*kdv*_ and *n*_*kdv*_ = 1 + *ϕ*_*kdv*_, where *x*_4_ is the initial phase of the KdV solitary wave. The parameters of two colliding waves are *ε*_1_ = 0.1, *ε*_2_ = 0.025, *k*_*d*_ = 1.0, *k*_*n*_ = 0.1, *μ* = 1.1, *ν* = 0.1, *β* = 0.1, *x*_3_ = *LX*/2 = 1200, *x*_4_ = *LX*/4 = 600, Δ*x* = 0.3, Δ*t* = Δ*x*/100, *N*_*x*_ = 8000, while *ω* = 0.1, *u*_*s*_ = 0.985.

## Additional Information

**How to cite this article**: Zhang, H. *et al.* Head-on collision and overtaking collision between an envelope solitary wave and a KdV solitary wave in a dusty plasma. *Sci. Rep.* **6**, 21214; doi: 10.1038/srep21214 (2016).
